# Prospective observational study of ^177^Lu-DOTA-octreotate therapy in 200 patients with advanced metastasized neuroendocrine tumours (NETs): feasibility and impact of a dosimetry-guided study protocol on outcome and toxicity

**DOI:** 10.1007/s00259-018-3945-z

**Published:** 2018-03-01

**Authors:** Ulrike Garske-Román, Mattias Sandström, Katarzyna Fröss Baron, Lars Lundin, Per Hellman, Staffan Welin, Silvia Johansson, Tanweera Khan, Hans Lundqvist, Barbro Eriksson, Anders Sundin, Dan Granberg

**Affiliations:** 1 Department of Clinical Physiology, Sahlgrenska University Hospital, Gothenburg, Sweden; 2 Department of Surgical Sciences, Uppsala University, Uppsala, Sweden; 3 Department of Immunology, Genetics and Pathology, Uppsala University, Uppsala, Sweden; 4 Department of Medical Sciences, Uppsala University, Uppsala, Sweden

**Keywords:** PRRT, ^177^Lu-DOTA-octreotate, Neuroendocrine tumour, Dosimetry, Outcome, Toxicity

## Abstract

**Purpose:**

Peptide receptor radionuclide therapy in patients with neuroendocrine tumours has yielded promising results. This prospective study investigated the feasibility of dosimetry of the kidneys and bone marrow during therapy and its impact on efficacy and outcome.

**Methods:**

The study group comprised 200 consecutive patients with metastasized somatostatin receptor-positive neuroendocrine tumours progressing on standard therapy or not suitable for other therapeutic options. A treatment cycle consisted of 7.4 GBq ^177^Lu-DOTA-octreotate with co-infusion of a mixed amino acid solution, and cycles were repeated until the absorbed dose to the kidneys reached 23 Gy or there were other reasons for stopping therapy. The Ki-67 index was ≤2% in 47 patients (23.5%), 3–20% in 121 (60.5%) and >20% in 16 (8%).

**Results:**

In 123 patients (61.5%) the absorbed dose to the kidneys reached 23 Gy with three to nine cycles during first-line therapy; in no patient was a dose to the bone marrow of 2 Gy reached. The best responses (according to RECIST 1.1) were a complete response (CR) in 1 patient (0.5%), a partial response (PR) in 47 (23.5%), stable disease (SD) in 135 (67.5%) and progressive disease (PD) in 7 (3.5%). Median progression-free survival was 27 months (95% CI 22–30 months) in all patients, 33 months in those in whom the absorbed dose to the kidneys reached 23 Gy and 15 months in those in whom it did not. Median overall survival (OS) was 43 months (95% CI 39–53 months) in all patients, 54 months in those in whom the absorbed dose to the kidneys reached 23 Gy and 25 months in those in whom it did not. Median OS was 60 months in patients with a best response of PR or CR, 42 months in those with SD and 16 months in those with PD. Three patients (1.5%) developed acute leukaemia, 1 patient (0.5%) chronic leukaemia (unconfirmed) and 30 patients (15%) grade 3 or 4 bone marrow toxicity. Eight patients (4%) developed grade 2 kidney toxicity and one patient (0.5%) grade 4 kidney toxicity.

**Conclusions:**

Dosimetry-based therapy with ^177^Lu-DOTA-octreotate is feasible. Patients in whom the absorbed dose to the kidneys reached 23 Gy had a longer OS than those in whom it did not. Patients with CR/PR had a longer OS than those with SD. Bone marrow dosimetry did not predict toxicity.

## Introduction

Peptide receptor radionuclide therapy (PRRT) has been used successfully in patients with neuroendocrine tumours (NETs) for two decades [1–5]. ^177^Lu-DOTA-octreotate [5–9], different ^90^Y–labelled peptides [10–14] and combinations of both have been used. ^177^Lu-DOTA-octreotate has usually been administered according to a standard protocol with four cycles of 7.4 GBq [5, 8, 9]. When PRRT became available in Sweden, the legal demand for individualized planning of radiotherapy necessitated the development of dosimetry procedures suitable for use under clinical conditions [15–20]. The organs at risk are the kidneys and bone marrow, with a growing body of data demonstrating higher nephrotoxicity with ^90^Y–labelled peptides using several schedules [11, 14, 21] than with ^177^Lu-DOTA-octreotate [5, 22–24]. The physical properties of lutetium-177 are well suited to following the distribution of the radionuclide for dosimetry during therapy. For ^177^Lu-DOTA-octreotate, a cumulative absorbed dose of 23 Gy to the kidneys was taken from external beam radiation [25], a cautious cut-off value in the setting of PRRT [26, 27]. A 2-Gy cut-off value for the bone marrow was based on experience with radioiodine therapy [28]. From dosimetry data compiled in our department we concluded that about 50% of patients might be undertreated and able to receive more than 4 × 7.4 GBq of ^177^Lu-DOTA-octreotate before reaching either 23 Gy to the kidneys or 2 Gy to the bone marrow [17].

The present study was designed to investigate the efficacy of treatment with ^177^Lu-DOTA-octreotate guided by dosimetry in patients with advanced NETs who either have progressive disease or are not suitable for standard care protocols. The primary aims were to evaluate the feasibility of individualized dosimetry in a routine setting, the applicability of dosimetry as a stop criterion for therapy aiming at an absorbed dose of 23 Gy to the kidneys and a maximum of 2 Gy to the bone marrow, and the side effects of PRRT performed under these conditions. Secondary endpoints were objective response according to RECIST 1.1 [29], progression-free survival (PFS) and overall survival (OS).

## Patients and methods

A total of 200 patients with NETs with somatostatin receptor expression higher than in normal liver (Krenning score 3 or 4 [30]) based on somatostatin receptor scintigraphy were enrolled after providing written informed consent (EudraCT no. 2009-012260-14). The local ethics and radiation ethics committees approved the study and the study was performed in accordance with the principles of the Declaration of Helsinki. Inclusion criteria were**:** metastatic NETs progressive on standard of care therapy, and proven intolerance or contraindications to other therapies. Patients with rectal NETs or bronchopulmonary carcinoids were accepted for first-line treatment, since no standard therapy has been established for metastatic disease. Life expectancy had to be more than 3 months, white blood cell count (WBC) >3.0 × 10^9^/L, platelet count >100 × 10^9^/L, bilirubin <40 μmol/L, albumin >25 g/L, creatinine <110 μmol/L or, if higher, glomerular filtration rate (cystatin-C) >50 ml/min/1.73 m^2^. Exclusion criteria were pregnancy, tumour amenable to surgery or radiofrequency ablation, and inability to stay isolated for 24 h. Of the 200 patients, 55% had small intestinal NETs (SI-NETs); for other tumour types see Table 1. After amendments to the initial study protocol, 16 patients (8%) diagnosed with grade 3 disease (Ki-67 index >20%) were accepted, and included those with a Ki-67 index up to 40%. All patients were receiving somatostatin analogues (SSA), and 43 (21.5%) had received higher than standard doses. SSA treatment was continued in all patients during PRRT. Patient characteristics are shown in Tables 1 and 2. The largest group of patients (84, 42%) were Swedish citizens; the remainder were referred from institutions in several other countries.

**Table 1:** Patient and tumour characteristics

Characteristic	Tumour type														Total
	SI-NET	Pancreatic NET			Rectal NET	NET, unknown origin	Lung carcinoid	Gastric NET	Neuroendocrine carcinoma	Paraganglioma	Phaeochromocytoma	Ovarian NET	Kidney NET	Duodenal NET	
		Total	Functioning	Nonfunctioning											
Number (%) of patients	108 (54)	48 (24)	19 (9.5)	29 (14.5)	11 (5.5)	8 (4)	6 (3)	5 (2.5)	5 (2.5)	3 (1.5)	2 (1)	2 (1)	1 (0.5)	1 (0.5)	200 (100)
Grade, *N* (%)^a^															
1	39 (36)	2 (4)	1 (5)	1 (3)	2 (18)	1 (12.5)	1 (17)	1 (20)	0 (0)	1	0 (0)	0 (0)	0 (0)	0 (0)	47 (23.5)
2	52 (48)	39 (81)	16 (84)	23 (79)	9 (82)	7 (87.5)	5 (83)	4 (80)	0 (0)	1	0 (0)	2 (100)	1 (100)	1 (100)	121 (60.5)
3	3 (3)	5 (10)	1 (5)	4 (14)	0 (0)	0 (0)	0 (0)	0 (0)	5 (100)	1	2 (100)	0 (0)	0 (0)	0 (0)	16 (8)
Unknown	14 (13)	2 (4)	1 (5)	1 (3)	0 (0)	0 (0)	0 (0)	0 (0)	0 (0)	0	0 (0)	0 (0)	0 (0)	0 (0)	16 (8)
Age (years), median (range)	66 (18–84)	58 (29–75)	63 (40–72)	58 (29–75)	60 (39–73)	65 (54–80)	69 (41–75)	61 (37–71)	58 (40–68)	65 (25–71)	55.7 (55–56)	72 (68–76)	47	76	63 (18–84)
Age >70 years, *N* (%)	32 (30)	5 (10)	2 (10.5)	3 (10)	1 (9)	2 (25)	3 (50)	1 (20)	0 (0)	1 (33)	0 (0)	1 (50)	0 (0)	1 (100)	47 (23.5)
Sex, *N* (%)															
Male	54 (50)	29 (60)	11 (58)	18 (62)	6 (56)	4 (50)	5 (83)	1 (20)	2 (40)	1	1	0	1	0	104 (52)
Female	54 (50)	19 (40)	8 (42)	11 (38)	5 (44)	4 (50)	1 (17)	4 (80)	3 (60)	2	1	2	0	1	96 (48)
Extensive disease, *N* (%)	47 (44)	18 (37.5)	11 (58)	7 (24)	9 (82)	7 (87.5)	2 (33)	4 (80)	2 (40)	0 (0)	0 (0)	1 (50)	1 (100)	0 (0)	91 (45.5)
Metastases, *N* (%)															
Liver	105 (97)	47 (98)	19 (100)	28 (97)	10 (91)	7 (87.5)	6 (100)	5 (100)	5 (100)	1 (33)	1 (50)	2 (100)	1 (100)	1 (100)	192 (96)
Lymph nodes	92 (85)	30 (62.5)	8 (42)	22 (76)	8 (73)	7 (87.5)	5 (83)	3 (60)	2 (40)	1 (33)	1 (50)	2 (100)	0 (0)	0 (0)	152 (76)
Pleura and peritoneum	41 (38)	3 (6)	1 (5)	2 (7)	2 (18)	0 (0)	1 (17)	1 (20)	0 (0)	0 (0)	0 (0)	1 (50)	0 (0)	1 (100)	50 (25)
Bone and bone marrow	53 (49)	16 (33)	7 (37)	9 (31)	10 (91)	6 (75)	6 (100)	4 (80)	0 (0)	3 (100)	2 (100)	2 (100)	1 (100)	0 (0)	103 (51.5)
Progression prior to inclusion, *N* (%)	87 (81)	44 (92)	18 (95)	26 (90)	7 (64)	6 (75)	4 (67)	4 (80)	3 (60)	1 (33)	1 (50)	2 (100)	1 (100)	1 (100)	161 (80.5)

^a^Grades 1, 2 and 3: Ki-67 index ≤2%, 3–20% and >20%, respectively

**Table 2:** Therapy prior to PRRT by tumour type. Complete data on previous therapies were available for 195 patients

Treatment before PRRT	Small intestine (*N* = 104)	Pancreas and duodenum (*N* = 49)	Rectum (*N* = 11)	Lung (*N* = 6)	Neural crest (*N* = 5)^a^	Other specified (*N* = 12)	Unknown primary (*N* = 8)	Total (*N* = 195)
Surgery								
Primary	77.9	36.7	36.4	33.3	60	50	0	59.1
Any type	80.8	36.7	45.4	33.3	100	50	12.5	62.2
Chemotherapy								
Streptozotocin/5-fluorouracil	1	77.6	18.2	16.7	0	0	62.5	23.9
Platinum-based	2.9	6.1	0	0	0	25	12.5	5.1
Temozolomide	1.9	44.9	0	50	0	33.3	37.5	15.3
Other	4.8	38.8	0	16.7	0	16.7	37.5	15.2
Total	8.7	100	18.2	66.7	0	58.3	87.5	39.6
Targeted therapies	4.8 (Ev *N* = 5; Sor, Bev, Sun *N* = 1)	26.5 (Ev *N* = 13; Sor, Bev, Sun *N* = 1)	9.1% (Ev, Im, Bev *N* = 1)	0	0	25 (Ev, Bev *N* = 2; Sun *N* = 1)	0	Ev 10.3; Bev 2.6; Sor, Sun, Im 0.5; Total 11.3
Biotherapy								
Somatostatin analogue	100	44.9	30	20	0	58.3	62.5	73.3
Interferon-α	47.1	6.1	0	0	0	0	0	26.7
Radiotherapy	4.8	6.5	10	16.7	0	16.7	37.5	7.9

The values are percentages

*Ev* everolimus, *Sor* sorafenib, *Bev* bevacizumab, *Sun* sunitinib, *Im* imatinib

^a^Neural crest(-derived) tumours: phaeochromocytoma and paraganglioma

Patients were treated with 7.4 GBq ^177^Lu-DOTA-octreotate per cycle with an intended interval of 6 to 8 weeks. The peptide was a kind gift from Prof. Eric Krenning. Lutetium-177 was purchased from IDB, Holland BV, and labelling was performed in-house. Kidney protection was provided by infusion of 2 L of an amino acid mixture (Vamin, 14 g N/L, electrolyte-free; Kabi Fresenius) over 8 h, starting half an hour before infusion of the radiopeptide. One hour before therapy, 8 mg of betamethasone and 8 mg of ondansetron or 250 μg palonosetron were given intravenously as antiemetic. Before every treatment cycle, WBC had to be >3 × 10^9^/L, granulocytes >1.5 × 10^9^/L and platelets >100 × 10^9^/L. Therapy was terminated if these criteria were not met within 6 months. As an exception, in some patients the activity was decreased by 30% instead of delaying treatment.

Cycles of 7.4 GBq ^177^Lu-DOTA-octreotate were repeated until the absorbed dose to the kidneys reached 23 Gy or there were other reasons for stopping therapy (Table 3). A cumulative absorbed dose of 2 Gy to the bone marrow was intended to act as a stop criterion, but was not reached in any of the patients during the initial treatment. In 14 patients (7%) with a favourable tumour response, salvage therapy aiming at a cumulative absorbed dose to the kidneys of 45 Gy was offered upon progression, given normal bone marrow and kidney function.

**Table 3:** Characteristics of patients who stopped therapy for bone marrow-related reasons or for other reasons

	Bone marrow-related reasons (*N* = 44, 22%)	Other reasons (*N* = 156, 78%)
Number of cycles of 7.4 GBq in initial treatment, median (range)	3.5 (1–6)	5 (1–10)
Bone marrow dose (Gy), median (range)		
First cycle		
Self-dose	0.0715 (0.049–0.133)	0.07 (0.033–0.321)
Total dose	0.138 (0.071–0.377)	0.136 (0.056–0.507)
First treatment (cumulative)		
Self-dose	0.281 (0.049–0.545)	0.3505 (0.06–0.976)
Total dose	0.4695 (0.071–0.995)	0.5505 (0.07–1.773)
Moderate or extensive skeletal disease, *N* (%)	9 (20.5)	26 (17)
Previous therapy, *N* (%)		
Chemotherapy	18 (41)	61 (39)
Targeted therapy, *N* (%)	2 (4.5)	20 (13)
Biotherapy		
SSA	36 (82)	110 (70.5)
Interferon-α	11 (25)	41 (26)
Intolerance of previous therapy, *N* (%)	12 (29)	44 (30)
Sex, *N* (%)		
Male	20 (45)	84 (54)
Female	24 (55)	72 (46)
Reasons for stopping therapy, *N* (%)		
Pancytopenia	18 (9)	–
Isolated thrombocytopenia	10 (5)	–
Progression and/or death after delay due to cytopenia	8 (4)	–
Combination of factors	8 (4)	4 (2)
Absorbed dose to the kidneys 23 Gy	–	114 (57)
Progression and/or death	–	18 (9)
Patient deteriorated/unable to cooperate	–	11 (5.5)
Progression after delay	–	3 (1.5)
Patient choice	–	2 (1)
Decrease in tumours to <10% of baseline	–	2 (1)
New SRS-negative tumours	–	2 (1)

Dosimetry for solid organs and bone marrow was performed as previously described in detail [15–17]. For solid organs, dosimetry was based on the small volume method performed on single photon emission tomography with low-dose CT (SPECT/CT) at 1, 4 and 7 days after therapy. Volumes of 4 cm^3^ were drawn on representative regions with homogeneous uptake. The activity concentrations were fitted to a monoexponential function. To calculate absorbed doses to the kidneys, the time-integrated activity concentrations were multiplied by the appropriate dose concentration factor considering only self-dose:

$$ {D}_O={DCF}^{\ast }{C}_{cumO} $$

where *D*_O_ is the absorbed dose to the organ, and DCF is the dose concentration factor converting *C*_cumO_ to absorbed dose by self absorption. Similarly, doses to the liver, spleen and representative tumours were calculated (data not shown). For complete bone marrow dosimetry, the dose from the blood activity (self-dose) was calculated from integrated blood activity curves derived from blood samples obtained at 0.5, 1, 2.5, 4, 8 and 24 h. This self-dose derived from the blood was complemented with the photon dose from resident activity in tumours, organs and the remainder of the body in order to calculate the total bone marrow dose. The photon dose was based on consecutive whole-body scans (WBS) at 1, 4 and 7 days after therapy. Regions of interests delineating the kidneys, liver, spleen, tumours and the whole body were drawn on geometrical mean images at 24 h and transferred to the images at 4 and 7 days. For calculation of the absorbed dose to bone marrow, the contribution of other source organs was added to the self-dose derived from the blood measurements as follows:

$$ {D}_{BM}={DCF}^{\ast }{C}_{cumBM}+\sum {DF_{BM\leftarrow T}}^{\ast }{A}_{cumT} $$

where *D*_BM_ is the absorbed dose to the bone marrow, *C*_cumBM_ is the time-integrated activity concentration in the bone marrow, DCF is the factor converting *C*_cumBM_ to absorbed dose by self-absorption, *A*_cumT_ is the time-integrated activity in tissue T (solid organs, tumour and the remainder of the body), and DF_BM ← T_ is the factor converting *A*_cumT_ to absorbed dose in the bone marrow (cross-fire).

Complete dosimetry was always performed during the first treatment, whenever large changes in tumour volume had occurred, after delay, and at least every fourth cycle. A short dosimetry protocol was performed for all other cycles after 24 h including a SPECT/CT scan over the abdomen and one WBS. The absorbed dose to the kidney was then calculated assuming an unchanged effective half-life, the amplitude for the area under the curve was adjusted for the actual measured activity concentration derived from the SPECT/CT scan at 24 h [16]. The self-dose from blood and the total bone marrow dose were calculated for each treatment with complete dosimetry and extrapolated for the remaining cycles by assuming the higher figure of two measurements for the adjacent unknown values. Radiological response was assessed using RECIST 1.1 criteria [29]. Whenever available, the biochemical response of biomarkers was monitored.

### Follow-up, data collection and statistics

Study enrolment was between September 2010 and February 2014. For patients from Sweden and Oslo (72.5%) survival data were derived from the respective national health registries that were accessed until May 2016. The referring institutions supplied all other follow-up data.

Nephrotoxicity, hepatotoxicity and bone marrow toxicity were recorded according to WHO criteria. Time to progression was calculated from the start of therapy to the date of radiologically confirmed progression (RECIST 1.1) except in clinically clear cases of progression based on scintigraphy, tumour markers and/or ultrasonography when CT data were not available (ten patients). OS was calculated based on all deaths that occurred until May 2016. Statistical evaluations including survival analysis using parametric Weibull plots and Kaplan-Meier curves were performed with the JMP 12.0.1 software package (SAS Institute Inc., Cary, NC). Differences between groups were assumed to be significant at *p* < 0.05 and were calculated using the log-rank, Wilcoxon, Student’s *t* and Pearson’s chi-squared tests where applicable. All analyses were performed on an intention-to-treat basis; all patients were included in the survival analysis. Time to progression was not reported for one patient who was excluded from the PFS calculation.

## Results

The dosimetry protocol applied was feasible. All patients underwent initial dosimetry. Most patients were able to leave hospital after 1 day, with patients who had to travel long distances staying in a hotel during weekdays when complete dosimetry was performed.

### Dosimetry

An absorbed dose of 23 Gy to the kidneys was reached after three to nine cycles of 7.4 GBq ^177^Lu-DOTA-octreotate. During the initial treatment, 98 patients (49%) received more than four (five to ten) cycles of 7.4 GBq, with a maximum of 74 GBq. One patient with pancreatic NET received a tenth cycle upon reaching the 23 Gy level after a favourable response in order to reach a tumour burden qualifying him for surgery of his primary tumour, which was performed several months after the last cycle. He had no signs of haematological or kidney toxicity. Patients in whom the dose to the kidneys reached 23 Gy received one to five cycles. Accumulated absorbed doses to the bone marrow ranged from 0.07 to 1.77 Gy for the complete treatment and from 0.07 to 0.51 for the first single cycle (Table 3). Fifty patients received exactly four cycles. The absorbed dose to the kidneys reached 23 Gy in 33 patients. Therapy was discontinued in 17 patients for other reasons, in six due to bone marrow suppression, in one due to tumour shrinkage by more than 90%, and in all others due to progression, clinical deterioration or death.

### Reasons for stopping therapy

First-line therapy was stopped upon reaching 23 Gy absorbed dose to the kidneys in 123 patients (61.5%), and in 114 of these this was the only stop criterion. The cumulative absorbed dose to the bone marrow did not reach 2 Gy in any patient. In 44 patients (22%) bone marrow-related toxicity was the only stop criterion. All reasons for stopping therapy are listed in Table 3.

### Objective responses according to RECIST 1.1

**Fig. 1:**
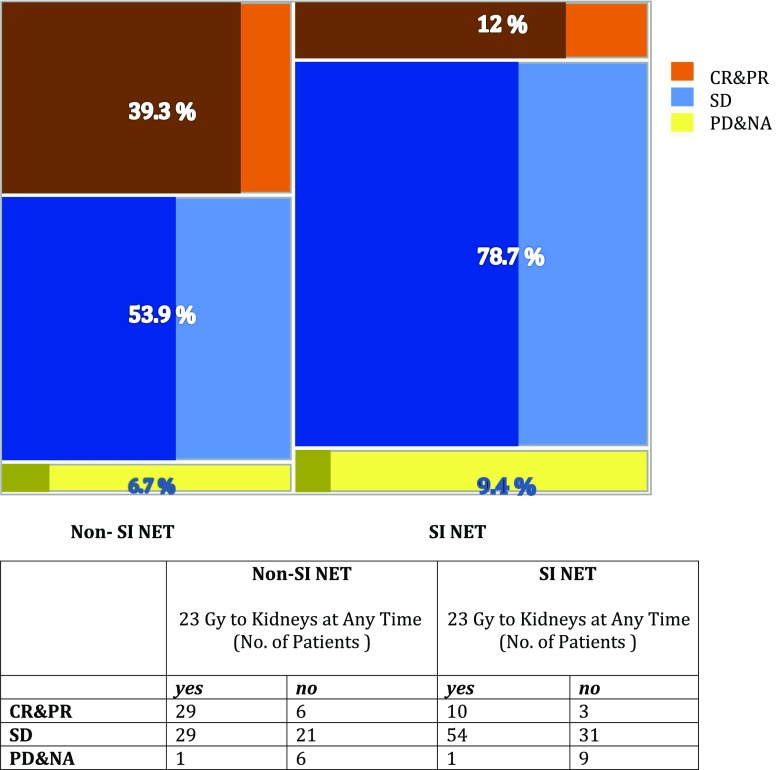
Patients with small intestinal NET (SI NET) show lower objective response rates according to RECIST 1.1 than patients with all other types of tumours (Non-SI NET). The proportions of patients in the respective groups with complete and partial responses (CR/PR), stable disease (SD) and progressive disease (PD) or data not available for radiological evaluation (NA) are indicated. *Shaded areas* Proportions of patients in whom the absorbed dose to the kidneys reached 23 Gy (numbers of patients are given in Table 4)

Tumour response varied among NET types (Table 4). Patients with SI-NETs showed a significantly lower objective response rate than patients with pancreatic and rectal NETs. Figure 1 illustrates the difference in objective response rates between patients with SI-NET and those with non-SI-NET. The proportions of patients in both groups in whom the absorbed dose to the kidneys reached 23 Gy are indicated. Of patients in whom the dose to the kidneys reached 23 Gy, 30.9% obtained an objective response (complete response and partial response, CR/PR) compared with 13% of patients in whom the dose to the kidneys did not reach 23 Gy for any reason (*p* < 0.0001). The response rate of 10.6% in patients with tumours with a low proliferation rate (Ki-67 index ≤2%) was lower than in patients with higher proliferation rates (30.6% and 31.3% for Ki-67 index 3–20% and >20%, respectively).

**Table 4:** Best response according to RECIST 1.1 in relation to tumour type

Tumour type	Number (%)	Best response, *N* (%)				
		Complete response	Partial response	Stable disease	Progressive disease	Not available
Total	200 (100)	1 (0.5)	47 (23.5)	135 (67.5)	7 (3.5)	10 (5)
SI-NET	108 (54)	0 (0)	13 (12)	85 (78.7)	2 (1.9)	8 (7.4)
Pancreatic and duodenal NET						
Total	49 (24.5)	1 (2)	21 (42.9)	24 (49)	2 (4.1)	1 (2)
Functioning	20 (10)	1 (5)	8 (40)	11 (55)	0 (0)	0 (0)
Nonfunctioning	29 (14.5)	0 (0)	13 (44.8)	13 (44.8)	2 (6.9)	1 (3.4)
Rectal NET	11 (5.5)	0 (0)	5 (45.5)	5 (45.5)	1 (9.1)	0 (0)
Unknown origin	8 (4)	0 (0)	3 (37.5)	4 (50)	1 (12.5)	0 (0)
Lung carcinoid	6 (3)	0 (0)	1 (16.7)	5 (83.3)	0 (0)	0 (0)
Gastric NET	5 (2.5)	0 (0)	1 (20)	2 (40)	1 (20)	1 (20)
Neuroendocrine carcinoma	5 (2.5)	0 (0)	2 (40)	3 (60)	0 (0)	0 (0)
Neural crest tumours^a^	5 (2.5)	0 (0)	0 (0)	5 (100)	0 (0)	0 (0)
Other	3(1.5)	0 (0)	1 (33.3)	2 (66.7)	0 (0)	0 (0)

^a^Phaeochromocytoma and paraganglioma

### Biochemical responses

Tumour markers including chromogranin A were available for analysis in 85 patients. A decrease of more than 50% in initially elevated levels occurred in 67% of patients, and was associated with a longer survival (median OS 60 months, 95% CI 42 months, upper limit not reached, NR) than a decrease of ≤50% (28 patients, 33%, median OS 35 months, 95% CI 17–52 months; *p* = 0.03). Tumour markers decreased by 50% or more in 80% of patients in whom the absorbed dose to the kidneys reached 23 Gy, but in only 45% of those in whom it did not (*p* = 0.0011). Of patients with an objective response (CR/PR), 91.3% (21 of 23) showed a decrease in tumour markers of more than 50%, in contrast to 62.5% of patients (35 of 56) with SD and 16.7% of nonresponders (1 of 6).

### Overall and progression-free survival

**Fig. 2:**
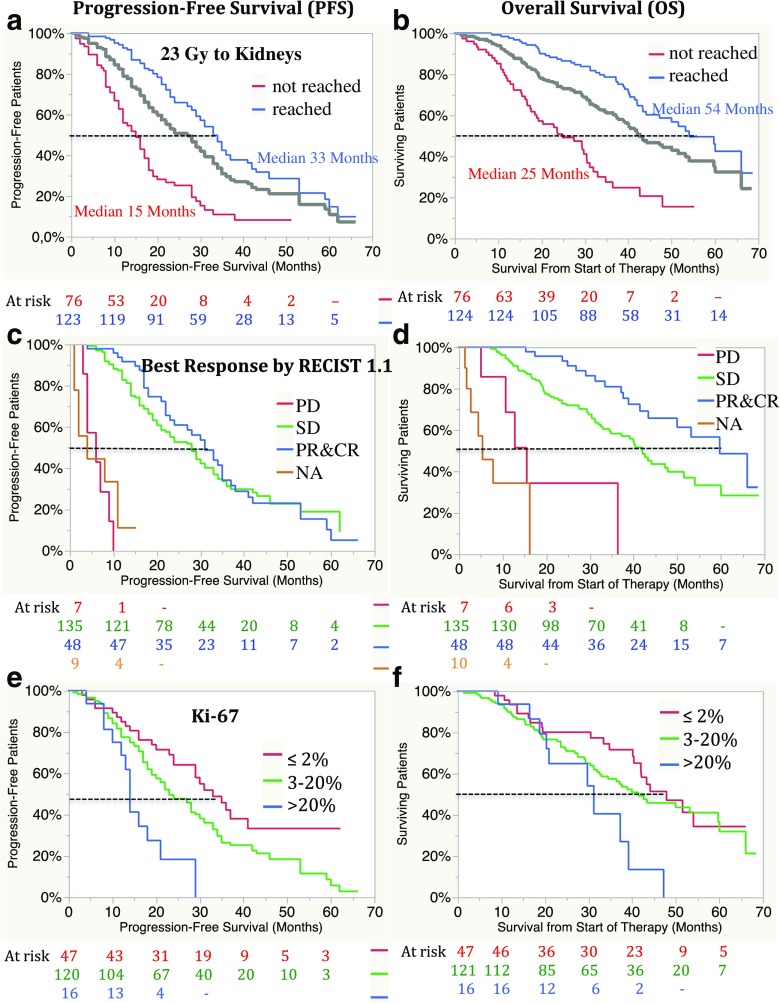
Progression-free survival (PFS; **a**, **c**, **e**) and overall survival (OS; **b**, **d**, **f**) from the start of therapy with ^177^Lu-DOTA-octreotate in relation to absorbed dose to the kidneys, best morphological response according to RECIST 1.1 and proliferation rate (Ki-67 index). **a**, **b** PFS (**a**) and OS (**b**) in 123 of 124 patients in whom the absorbed dose to the kidneys reached 23 Gy (*blue line*) and in 76 patients in whom it did not (*red line*); *grey line* combined data. **c**, **d** PFS (**c**) and OS (**d**) in relation to best response according to RECIST 1.1: progressive disease (PD, *red line*), median PFS 6 months (95% CI 3–9 months), median OS 15.5 months (95% CI 5–36 months); stable disease (SD, *green line*), median PFS 28 months (95% CI 21–31 months), OS 42 months (95% CI 34–51.5 months); partial response/complete response (PR&CR, *blue line*), median PFS 31 months (95% CI 23–35 months), OS 60 months (95% CI 43 months, upper limit not reached, NR). **e**, **f** PFS (**e**) and OS (**f**) in relation to proliferation rate (Ki-67 index): grade 1, Ki-67 index ≤2% (*red line*), PFS 33 months (95% CI 24–41 months), OS 48 months (95% CI 40 months, NR); grade 2, Ki-67 index 3–20% (*green line*), median PFS 23 months (95% CI 19–28 months), median OS 41 months (95% CI 32.5–60 months); grade 3, Ki-67 index >20% (*blue line*), median PFS 14 months (95% CI 10–21 months), median OS 31 months (95% CI 19–39 months)

Median follow-up was 31 months (range 1–68 months). Of the 200 patients, 106 (53%) were alive at the time of analysis, and of these, 29% were progression-free. The main cause of death was tumour progression (65 of 94 patients, 70%). In 16 patients (15%), the cause of death was unknown. Median OS was 43 months (95% CI 39–53 months) in all patients. Median OS in the patients in whom the absorbed dose to the kidneys reached 23 Gy was 54 months (95% CI 44 months, NR) and 25 months (95% CI 18–30 months) in those in whom it did not (*p* < 0.0001; Fig. 2, Table 5).

**Table 5:** Progression-free survival (PFS) and overall survival (OS) in relation to tumour type and whether the absorbed dose to the kidneys did or did not reach 23 Gy

Tumour type	Number (% of total)	23 Gy to the kidneys reached (any time), *N* (%)^a^	Progression-free survival, median (95% CI)^b^	Overall survival, median (95% CI)^c^
Total	200 (100)	Yes 124 (62)	33 (29–36)*	54 (44, NR)*
		No 76 (38)	15 (12–17)*	25 (18–30)*
		All 200 (100)	27 (22–30)	43 (39–53)
SI-NET	108 (54)	Yes 65 (60.2)	42 (33, NR)*	60 (44, NR)*
		No 43 (39.8)	18 (13–22)*	29 (17–35)*
		All 108 (100)	29 (23–35)	48 (40–60)
Pancreatic and duodenal NET				
Total	49 (24.5)	Yes 31 (63.3)	33 (27–38)*	53 (41, NR)*
		No 18 (36.7)	10.5 (8–14)*	24 (10–29)*
		All 49 (100)	27 (17–33)	42 (31, NR)
Functioning	20 (10)	Yes 11 (55)	33 (17–46)*	42 (37, NR)*
		No 9 (45)	12 (8–38)*	23 (9–29)*
		All 20 (100)	24 (12–37)	39 (24–53)
Nonfunctioning	29 (14.5)	Yes 19 (65.5)	31 (23–46)*	NR (38, NR)*
		No 10 (34.5)	10.5 (4–17)*	27 (5, NR)*
		All 29 (100)	27 (14–33)	NR (31, NR)
Rectal NET	11 (5.5)	Yes 8 (72.7)	34 (17–35)*	50 (33, NR)*
		No 3 (27.3)	12 (3–12)*	12 (11–33)*
		All 11 (100)	33 (12–35)	34 (12, NR)
Unknown origin	8 (4)	Yes 3 (37.5)	34 (16–41)	NR (20, NR)
		No 5 (62.5)	17 (7–28)	31 (15–43)
		All8 (100)	17.5 (7–34)	43 (15, NR)
Bronchopulmonary carcinoid	6 (3)	Yes 5 (83.3)	20 (14–43)	
		No 1 (16.7)	12	
		All 6 (100)	18 (12–43)	NR (19, NR)
Gastric NET	5 (2.5)	Yes 1 (20)		
		No 4 (80)		
		All 5 (100)	12 (8–16)	17 (12–36)
Neuroendocrine carcinoma	5 (2.5)	Yes 3 (60)		
		No 2 (40)		
		All 5 (100)	14 (4–29)	30 (21–47)
Neural crest tumours	5 (2.5)	Yes 5 (100)		
		No 0 (0)		
		All 5 (100)	14 (12, NR)	37 (16–54)
Others	3 (1.5)	Yes 3 (100)		
		No 0 (0)		
		All 3 (100)	30 (21–53)	43 (39–53)

*NR* upper limit not reached

*p < 0.05, between groups

^a^In one patient the absorbed dose to the kidneys reached 23 Gy at the time of salvage therapy

^b^PFS was calculated on the basis of whether a dose of 23 Gy was reached during first-line treatment (123 patients)

^c^OS was calculated on the basis of whether a dose of 23 Gy was reached at any time (124 patients)

**Fig. 3:**
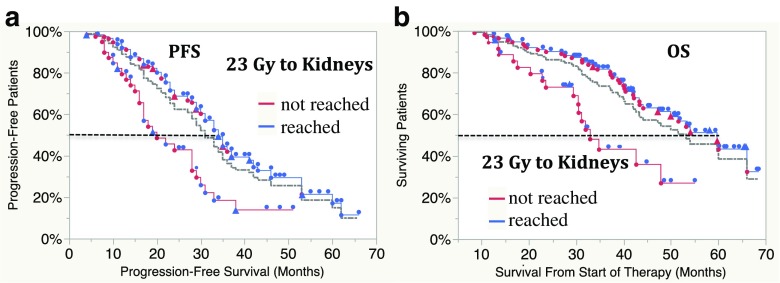
Progression-free survival (PFS, **a**) and overall survival (OS, **b**) in 154 patients who stopped therapy for reasons other than progression or clinical deterioration. In 114 patients in whom the absorbed dose to the kidneys reached 23 Gy, PFS was 34 months (95% CI 31–37 months) and OS was 60 months (95% CI 47 months, NR). In 39 of 40 patients in whom a dose of 23 Gy was not reached, PFS was 20 months (95% CI 16–28 months) and OS was 33 months (95% CI 29–48 months; *p* < 0.0001 for PFS, *p* = 0.0004 for OS); *grey line* combined data, *red symbols* patient died, *blue symbols* patient alive, *triangles* patient received salvage therapy

**Fig. 4:**
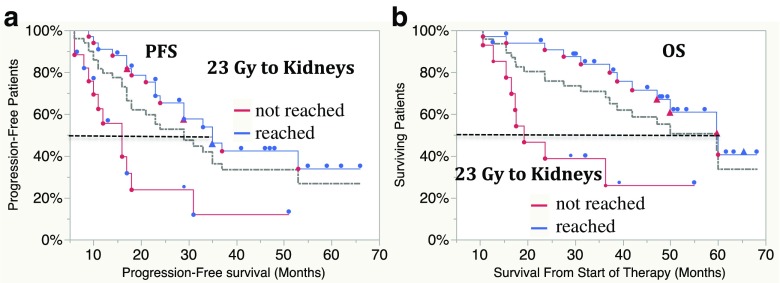
Progression-free survival (PFS, **a**) and overall survival (OS, **b**) in all 50 patients who received exactly four cycles of ^177^Lu-DOTA-octreotate. In 33 patients in whom the absorbed dose to the kidneys reached 23 Gy, PFS was 35 months (95% CI 23 months, NR) and OS was 60 months (95% CI 47 months, NR). In 17 patients in whom a dose of 23 Gy was not reached, PFS was 16 months (95% CI 9–18 months) and OS was 19 months (95% CI 15 months, NR; *p* = 0.0003 for PFS, *p* = 0.0006 for OS, log-rank test; *grey line* combined data, *red symbols* patient died, *blue symbols* patient alive, *triangles* patient received salvage therapy

Median PFS was 27 months (95% CI 22–30 months) in all patients, 33 months (95% CI 29–36 months) in patients in whom the absorbed dose to the kidneys reached 23 Gy during the initial treatment, and 15 months (95% CI 12–17 months) in those in whom it did not (*p* < 0.0001). In order to compensate for bias due to therapy discontinuation related to progression or deterioration of the patient during therapy, PFS and OS were also calculated after exclusion of 46 patients who stopped therapy for these reasons (Fig. 3). In the remaining 154 patients, PFS and OS were 34 and 60 months in those in whom the absorbed dose to the kidneys reached 23 Gy and 20 and 33 months in those in whom it did not, respectively (*p* = 0.001 for PFS, *p* = 0.0004 for OS). A subgroup analysis of patients who received exactly four cycles of ^177^Lu-DOTA-octreotate is shown in Fig. 4. OS, but not PFS, was significantly longer in patients with an objective response (CR/PR) than in patients with SD (median OS 60 months, 95% CI 43 months, NR, versus 42 months, 95% CI 34–52 months, *p* = 0.004; median PFS 31 months, 95% CI 23–35 months, versus 28 months, 95% CI 21–31 months, not significant; Fig. 2, Table 5). Patients with a lower Ki-67 index had longer PFS (Fig. 2).

### Toxicity, side effects and serious adverse events

In 69 patients (34.5%), one or several cycles had to be delayed due to thrombocytopenia and/or leucopenia, alone or in combination with anaemia (Table 3). Grade 3 or 4 bone marrow toxicity of any kind was seen in 30 (15%) of the 200 patients. Bone marrow toxicity was generally transient and therapy was continued after the nadir had passed. The treatment was stopped in 44 patients (22%) for bone marrow-related reasons, including 5 patients with tumour progression while awaiting bone marrow recovery.

Grade 1 nephrotoxicity at any time was seen in 38 patients (19%), most of whom were stable or improved during therapy. Grade 2 nephrotoxicity was seen in 8 patients (4%), all but one with concomitant risk factors (arterial hypertension, cardiac insufficiency and/or diabetes mellitus). Grade 2 toxicity was seen in all patients at the time of progression during general deterioration prior to death. No grade 3 toxicity was seen. Grade 4 nephrotoxicity was seen in one female patient 3 years after therapy. This patient had a history of arterial hypertension, and treatment was stopped after cycle 2 because of an unexplained increase in the absorbed kidney dose from 6.1 to 9.1 Gy (right) and 6.4 to 9.6 Gy (left), resulting in cumulative absorbed doses of 15.2 to the right kidney and 16 Gy to the left kidney. Hypertensive nephrosclerosis was confirmed histologically.

Six patients died from cardiac events (infarction or progressive carcinoid heart failure) and four patients died from infections, two of them with cytopenic fever. Two patients developed and subsequently died from acute leukaemia, one myeloid (AML) and one lymphoblastic (ALL). One patient developed massive leucocytosis following a jaw infection and chronic myeloid leukaemia (CML) was suspected. The patient died due to the underlying infection before a final diagnosis could be established. One patient developed AML and was alive at the time of follow-up. This patient had previously received streptozotocin/capecitabine and temozolomide upon progression after PRRT. The patients with ALL and suspected CML had received temozolomide before PRRT. All four patients had bone marrow metastases. Leukaemia was diagnosed between 18 and 46 months after the start of PRRT.

## Discussion

To our knowledge, this is the first prospective study to explore the outcome of PRRT with ^177^Lu-DOTA-octreotate in patients with NET applying systematic, individualized dosimetry of the kidneys and bone marrow. The study provides evidence supporting our assumption that about half of the patients can tolerate more than four cycles of 7.4 GBq ^177^Lu-DOTA-octreotate [17]. The question to be answered is whether outcome is improved in the substantial proportion of patients who would receive a higher activity than given using the widely accepted Rotterdam protocol of four cycles of 7.4 GBq ^177^Lu-DOTA-octreotate. In Table 6, the number of cycles per patient during the initial treatment is given, along with information as to whether 23 Gy to the kidneys was reached and the number of surviving patients at the time of analysis.

**Table 6:** Number of cycles per patient given in the initial treatment for the largest tumour groups**.** The majority of patients (68.5%) received more than four cycles to reach an absorbed dose to the kidneys of 23 Gy. Of patients still living at the time of analysis, the majority (56.4%) received more than four cycles. Of patients who had died, a smaller proportion (43.6%) had received more than four cycles

	Number of cycles per patient of 7.4 GBq 177Lu-DOTA-octreotate in initial treatment										Total patients
	One	Two	Three	Four	Five	Six	Seven	Eight	Nine	Ten	
Tumour type											
SI-NET	5	8	16	28	24	19	7	0	1	0	108
Pancreatic and duodenal NET	0	3	9	13	7	10	5	0	0	1	48
Rectal NET	0	1	0	2	4	3	0	1	0	0	11
Unknown origin	0	1	1	2	1	1	2	0	0	0	8
Lung carcinoid	0	0	0	0	3	1	1	1	0	0	6
Total patients	5	13	26	45	39	34	15	1	2	1	181
23 Gy absorbed dose to the kidneys on initial treatment											
Yes	0	0	5	30	26	32	13	2	1	1	111 (≤4 cycles 35, 31.5%; >4 cycles 76, 68.5%)
No	5	13	21	15	13	2	2	0	0	0	71 (≤4 cycles 54, 76.1%; >4 cycles 17, 23.9%)
Alive											
Yes	0	5	10	29	20	26	9	1	1	0	101 (≤4 cycles 44, 43.6%; >4 cycles 57, 56.4%)
No	5	8	16	16	19	8	6	1	0	1	80 (≤4 cycles 45, 56.25%; >4 cycles 35, 43.75%)

Data so far published are not easily compared since the studies were inhomogeneous regarding tumour type, risk factors and previous therapy as well as endpoints. In the present study, several risk factors were more prevalent than in the majority of other larger studies (Table 7). In 8% of patients the Ki-67 index was >20% and in 29% >10%, a level found to affect survival [7]. PFS was significantly lower in patients with a Ki-67 index >20% (Fig. 2), but a median PFS of 14 months (95% CI 10–21 months) and a median OS of 31 months (95% CI 19–39 months) in our 16 patients still compares well with previous published data on patients with high-grade NETs [31, 32]. This confirms that patients with a high-grade tumour and sufficient somatostatin receptor expression may benefit from PRRT, as described in previous case reports [33–35]. In these patients, higher accumulated activities and an individualized protocol may be of special importance, and fractionation may partially explain improved tumour-to-background ratios as previously discussed [35]. The response rates in patients with a Ki-67 index >20% were higher than in those reported by Ezziddin et al., who aimed at four cycles of 7.9 GBq ^177^Lu-DOTATATE with an intended interval of 10 to 14 weeks [34]. Among their seven patients with a Ki-67 index >20%, only one had PR and SD (14%), respectively, while five patients (71%) progressed. In our study, 43.8% of patients received more than four (five to seven) cycles of 7.4 GBq with an intended interval of 6 to 8 weeks. PR was seen in 5 of 16 patients (31%) and SD in 69%, and no progression at first follow-up examination.

**Table 7:** Comparison of studies using ^90^Y-labelled or ^177^ Lu-labelled somatostatin analogues

	[10, 11]	[12]	[13]	[14]	[5]	[6]	[7]	Present study
Number of patients	58	90	60	1,109	310	51	74	200
Ligand	^90^Y-DOTA-TOC	^90^Y-edotreotide	^90^Y-DOTA-TATE	^90^Y-DOTA-TOC	^177^Lu-DOTA-TATE	^177^Lu-DOTA-TATE	^177^Lu-DOTA-TATE	^177^Lu-DOTA-TATE
Dosimetry	No; activity escalation	No; 3 × 4.4 GBq	<23 Gy kidney; 2 Gy bone marrow	No; 3.7 GBq/m^2^ body surface area, repeat based on toxicity	No; 4 × 7.4 GBq per cycle	Yes; activity escalation	No; 7.9 GBq × 4	Yes, all patients; 7.4 GBq per cycle up to 23 Gy absorbed dose to the kidneys
Cumulative activity	Maximum cumulative activity 14.9 GBq/m^2^ per cycle 0.7–3.8 GBq	Maximum cumulative activity 13.3 GBq (in one to three cycles)	Range 4.1–16.2 GBq (mean 11.2 GBq cumulative; 3.7 GBq/cycle)	3.7 GBq/m^2^ (one to ten cycles, median two)	Maximum cumulative activity 29.6 GBq (3.7–7.4 GBq per cycle)	Maximum cumulative activity 29.2 GBq (3.7–7.4 GBq/cycle)	Maximum cumulative activity 31.6 GBq	Range 5–74 GBq (mean 32.8, median 29.6; 7.4 GBq per cycle in 92.5% of all cycles (one to ten cycles per patient)
Liver metastases (%)	90	72.2	85	82.2	89	80.4	78.4	96
Bone/bone marrow metastases (%)	NA	18.9	11.6	19	22	21.6	37.8 (SRS)	51 (Lu-DOTATATE)
Extensive disease (%)	31 (“end-stage”)	NA	NA (80 stage 4)	NA	22 (SRS); 27 (CT/ MRI)	27.5 (liver only)	50 >25% liver involvement (SRS)	44 (39 >50% liver involvement) (Lu-DOTATATE)
Progression at start (%)	81	NA	100	100	43	79.6	75.7	80.5
Previous chemotherapy (%)	31	31.1	57	29.7	17	12.1	24.3	39.6
Ki-67 index >20% (%)	NA	NA	0	NA	NA	7.8	0	8
Ki-67 index >10% (%)	NA	NA	NA	NA	NA	NA	19.4	29
SI-NET (%)	52	NA	48	23.9	60.6	37.3	25.7	54
Median OS from start of therapy, months (95% CI)	36.7 (19.4–54.1)	26.9 (CI NA)	22 (20.6–26.7)	46 responders; 17 nonresponders	46 (CI NA)	NR	55 (48.8–61.2)	43 (39–53; 54 vs. 25 if 23 Gy to the kidneys reached)
Median PFS, months (95% CI)	14.3 (8.9–19.7)	16.3	17 (16.4–21.2)	NA	33	NA	26	29 (24–33; 35 vs. 17 if 23 Gy to kidneys reached)
Outcome								
Median follow-up (months)	NA	NA	NA	23	19	29 (range 4–66)	47	31
Response (%)								
Criteria	SWOG	SWOG	RECIST	(RECIST)	SWOG	(RECIST)	SWOG	RECIST 1.1
Complete response	0	0	0	0.6	2	2	0	0.5
Partial response	8.6	4.4	23	6.5	28	27	36.5	23.5
Minor response	12.1	NA	NA	NA	16	26	17.6	– (35.5 >10% decrease)
Stable disease	50	70.0	77	5.2	35	27	35.1	67.5 (32)
Progressive disease	24.2	12.2	0	39.5	20	18	10.8	3.5
Not available	5.2	13.3	0	NA	–	–	–	5
Median OS from start of therapy, months (95% CI)	36.7 (19.4–54.1)	26.9 (CI NA)	22 (20.6–26.7)	46 responders; 17 non-responders	46 (CI NA)	NR	55 (48.8–61.2)	43 (39–53; 54 vs. 25 if 23 Gy to the kidneys)
Median time to progression (months)	NA	NA	NA	12.7	40	36 (24–50)	NA	NA
Median PFS, months (95% CI)	14.3 (8.9–19.7)	16.3	17 (16.4–21.2)	NA	33	NA	26	27 (22–30; 33 vs. 15 if 23 Gy to the kidneys)
Side effects								
Bone marrow malignancy (%)	MDS 1.1	0	0	0.1 AML; 0.1 MDS	4/504 MDS (0.8%)	0	0	0.5 ALL, 1 AML, 0.5 CML (unconfirmed)
Haematotoxicity grade 3/4 (%)	1.1	17.2	8 WBC; 10 Hb; 2 Trbc	12.8	9.5	2	<10	15
Kidney toxicity grade 3/4/5(%)	2.2	3.3	5	9.2	2 of 504 (0.4%)	0	0	0.5

*SRS* somatostatin receptor scintigraphy, *NR* not reached, *BM* bone marrow, *OS* overall survival, *PFS* progression-free survival, *NA* not available, *MDS* myelodysplastic syndrome, *ALL* acute lymphoblastic leukaemia, *AML* acute myeloid leukaemia, *CML* chronic myeloid leukaemia, *Hb* haemoglobin, *Trbc* thrombocytes

Few observations of a dose-response relationship in PRRT have been reported [36, 37]. To prescribe tumour doses in PRRT, as in external beam radiation, is not possible, but the present results indicate that the absorbed dose to the kidneys can serve as a substitute for tumour dose and the tolerance of the individual patient. Bodei et al. found a positive correlation between objective response and cumulative activity, that was in turn related to higher absorbed tumour doses [6].

**Fig. 5:**
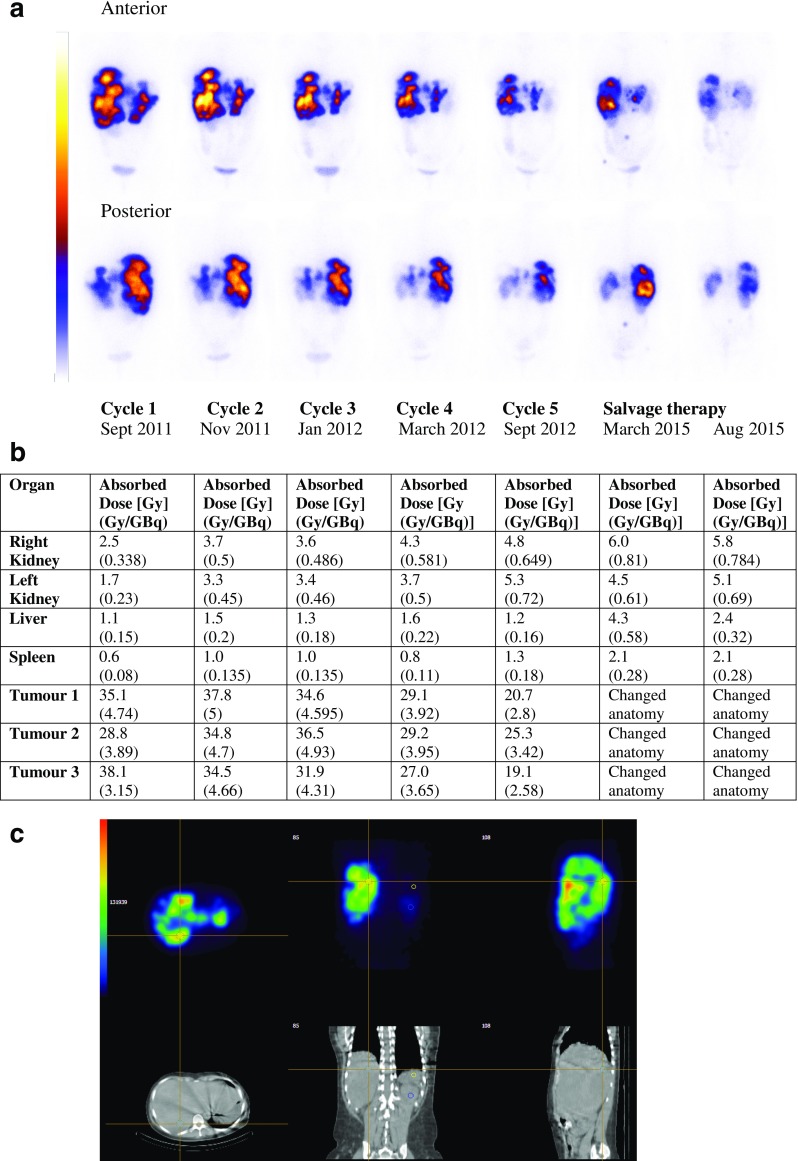
A 43-year-old female patient with VIPoma. **a** Cropped whole-body scans (WBS) obtained on day 1 after treatment with ^177^Lu-DOTA-octreotate during initial treatment (cycles 1–5) and salvage therapy. Note the change in size and uptake intensity of the different tumours throughout therapy. Imaging at the time of salvage therapy after progression shows new uptake in the skeleton, but the total tumour burden is still lower than at the start of therapy. **b** Absorbed organ doses (in absolute values and in relation to administered activity) to the kidneys, liver parenchyma and spleen , and to three representative tumour lesions with homogeneous uptake, measured in the same area during each cycle of initial treatment and during salvage therapy. **c** Layout of the dosimetry programme (implemented on a Hermes platform, Nuclear Diagnostics International AB). The hair cross is positioned on the metastatic uptake in the liver, the small circle corresponds to a 4-cm^3^ volume of interest (VOI). For tumour and solid organ dosimetry, calculations of absorbed doses were based on volume concentrations in small VOIs drawn on SPECT/CT images at three time-points (24 h, 4 days and 7 days after infusion). In this patient, three SPECT/CT scans were acquired during cycles 1, 2 and 4 and cycle 1 of salvage therapy. For the other cycles, one SPECT/CT scan was acquired at 24 h and the absorbed dose was calculated assuming unchanged kinetics from the previous therapy

The relationships among absorbed dose, objective tumour shrinkage and survival are not easy to determine in patients with NETs undergoing PRRT, since the time from the start of therapy to best response varies and can be several years [5], in our study up to 54 months. Based on best response, patients with CR or PR had a significantly longer OS than those with SD (Fig. 2). Kwekkeboom et al. found no significant difference in survival between patients with CR or PR and those with SD based on their radiological response 3 months after therapy [5], which may be attributable to the earlier time point for evaluation. In a recent update, the Rotterdam group have also reported longer survival in patients with CR or PR as best response than in those with SD [9]. On the other hand, we found no difference between these groups in terms of PFS. One explanation for this may be that at the time of progression, patients become amenable to further treatment after previous substantial tumour regression. An example of this is presented in Fig. 5 that shows the WBS findings in a patient with massive liver metastases of a VIPoma. After the first cycle of ^177^Lu-DOTA-octreotate, she developed a hormonal crisis with life-threatening diarrhoea requiring intensive care. After three cycles, vasoactive intestinal peptide levels normalized and the diarrhoea stopped completely. The best morphological result was a PR with a decrease by 45% according to RECIST 1.1 after cycle 5. By 40 months after the start of therapy, the tumour had progressed and salvage therapy was given with another two cycles. At this time-point, the tumour burden was still lower than at the start of therapy, and the patient’s general condition had improved. In the current series, 14 patients were able to receive salvage therapy, of whom 9 (64.2%) were alive at the time of analysis.

There is a relationship between objective responses and decrease of tumour markers, which is in turn related to OS, and according to Khan et al. [38] is also associated with improvement in quality of life. In this study, decreases in tumour markers by more than 50% from increased baseline levels in 65% of 85 patients were associated with longer survival with a median OS of 60 months (95% CI 42 months, NR) versus 35 months (95% CI 17–52 months; *p* = 0.01*).* Furthermore, more patients in whom the absorbed dose to the kidneys reached 23 Gy showed a decrease in tumour markers.

In agreement with other studies, in the present study the objective response rate was lower in patients with SI-NETs than in those with other NETs [4, 14], but the survival rate was not lower (Tables 4 and 5, Fig. 1). In the present study, 24% of patients obtained an objective response (RECIST 1.1 CR/PR), with better results in those with pancreaticoduodenal NETs (42.9%) and rectal NETs (45.4%) than in those with SI-NETs (12%). In the NETTER-1 study, four cycles of 7.4 GBq ^177^Lu-DOTA-octreotate was compared with an increased dose of SSA in a group of patients with grade 1 and grade 2 SI-NETs, and a higher objective response rate with PRRT (18.8% versus 3%) was found with an estimated PFS rate of 65.2% (95% CI 50–76.8%) at 20 months [8]. This is comparable to 68% (95% CI 59–75%) found in the present study (parametric Weibull fit, supported by Kaplan-Meier analysis), despite the fact that 31.5% of the patients had increased doses of SSA on enrolment and risk factors such as liver and bone metastases being more frequent in our study (liver metastases 84% versus 97%, bone metastases 11% versus 49%). Table 8 compares patient groups with similar inclusion characteristics from the NETTER-1 study [8], the recent update from the Rotterdam group (table adapted from Brabander et al. [9]) and the present study.

**Table 8:** Patient characteristics, overall response rates, progression-free survival at 20 months and overall survival in patients with small intestinal NET. Comparison between data from the NETTER-1 study, the recent update from the Erasmus group [9] and subgroups from the present study with comparable patient characteristics (grade 1 and grade 2, progressive at inclusion). For the present study, data are given separately for the patient group that had not received increased somatostatin receptor (SSR) analogues previously and the whole group of patients with progressive tumours grade 1 and 2 patients disregarding previous SSR analogue treatment (adapted after Brabander et al. [9])

	NETTER-1 study (*N* = 116) [8]	Erasmus group 2017 update (*N* = 106) [9]	Present study (grade 1 and 2, progressive)	
			Patients with no increase in SSR analogues (*N* = 24)	Patients with all levels SSR analogues (*N* = 76)
Sex, *N* (%)				
Female	53 (46)	52 (49)	12 (50)	36 (47)
Male	63 (54)	54 (51)	12 (50)	40 (53)
Age (years), mean ± SD	63 ± 9	62 ± 10	67 ± 8	66 ± 10
Site of metastases, *N* (%)				
Liver	97 (84)	97 (92)	24 (100)	75 (99)
Bone	13 (11)	14 (13)	11 (46)	38 (50)
Extent of disease, *N* (%)				
Limited	99 (85)	4 (4)	0 (0)	7 (9)
Moderate	13 (11)	82 (77)	12 (50)	38 (51)
Extensive	4 (3)	20 (19)	12 (50)	31 (41)
Previous treatment, *N* (%)				
Surgery	93 (80)	60 (57)	20 (83)	63 (83)
Chemotherapy	11 (9)	6 (6)	1 (4)	6 (8)
Radiotherapy	4 (3)	3 (3)	0 (0)	5 (7)
Previous SSR therapy, *N* (%)	116 (100)	89 (84)	24 (100)	73 (96)
Previous interferon-α, *N* (%)	NA	NA	10 (42)	35 (46)
Overall response rate, *N* (%)	18 (16)	29 (27)	6 (25)	11 (14)
Progression-free survival rate at 20 months (%)	65	58	72	67
Median overall survival (months)	NR	46	48 (95% CI 25, NR)	43 (95% CI 35–60)

*NR* upper limit not reached

**Fig. 6:**
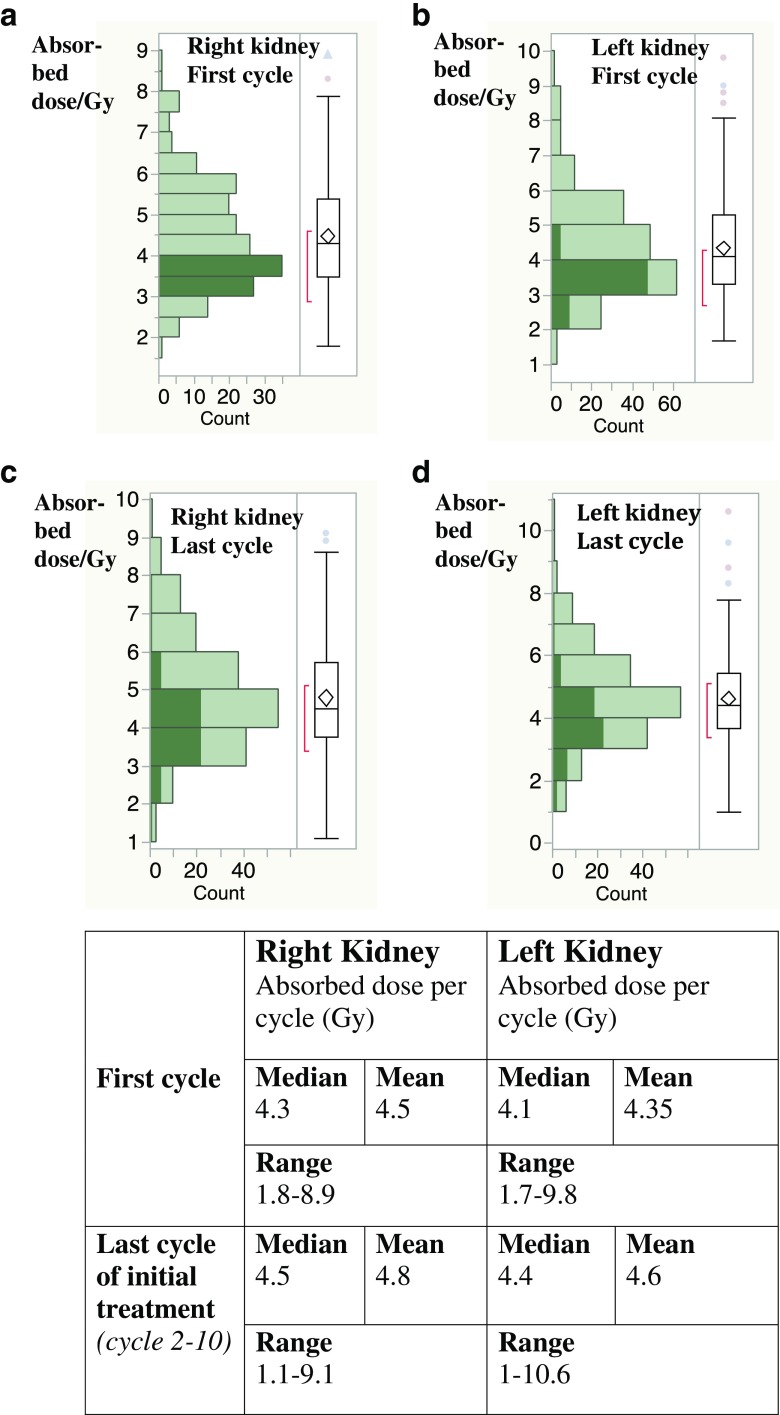
Absorbed doses to the kidneys in the first cycle (**a**, **b**) and the last cycle in each patient (cycles 2 to 10; **c**, **d**) during initial treatment. Throughout therapy, doses could decrease or increase. *Shaded bars* indicate patients who received an absorbed dose of 3 to 4 Gy to their right kidney in the first cycle (**a**). In their last cycle, absorbed doses to the right kidney varied between 2 and 6 Gy (**c**). Dose distributions in the left kidneys of the same patients correspond to shaded areas in **b** and **d**

We conclude that an absorbed dose of 23 Gy is generally well tolerated by all patients in whom this limit can be reached, regardless of the number of cycles. The only case of grade 4 nephrotoxicity observed was found to be related to hypertensive nephrosclerosis before this dose had been reached, at cumulative doses of 15.2 and 16 Gy to the right and left kidney, respectively. Therapy was stopped because of an increase of about 50% between cycle 1 and cycle 2, so that further damage was avoided. We interpreted this toxicity as primarily caused by the patient’s hypertension, but probably worsened by the treatment. A grade 2 kidney toxicity was seen in eight patients (4%) at the time of progression as part of general deterioration, and was probably unrelated to the treatment. and seven of these patients had cardiovascular risk factors and/or diabetes mellitus. On the other hand, risk factors for kidney toxicity (cardiovascular disease including arterial hypertension and diabetes mellitus) were present in 79 patients (39.5%), and in 43 of these patients (54%) the absorbed dose to the kidneys reached 23 Gy, with 37 of these 43 patients receiving more than four cycles (five to seven) without toxicity. This demonstrates that even in the presence of risk factors, the limit of 23 Gy to the kidneys is generally safe, and patients with risk factors need not automatically be excluded from the attempt to reach this goal with higher activities if they show clinical benefit. Kidney dosimetry can help reveal abnormal dose changes and levels and minimize damage. Figure 6 illustrates the range of absorbed doses to the kidneys that were observed per cycle and patient and the changes over time.

Even higher cumulative absorbed doses to the kidneys (with maximum absorbed doses to the right and left kidney of 46.6 and 43.3 Gy, respectively) were reached in 14 patients (7%) who underwent retreatment without nephrotoxicity worse than grade 1. This implies that higher absorbed doses from ^177^Lu-DOTA-octreotate may be possible in patients without risk factors. The tolerability of higher biological effective doses (BED) to the kidneys is currently being studied [39]. The relationship between absorbed doses (as reported in the current study) and BED is discussed by Cremonesi et al. [40] and in a recent report from our group [41].

The Rotterdam protocol of four cycles of 7.4 GBq ^177^Lu-DOTA-octreotate has been widely accepted. In patients who received four cycles during initial treatment, those in whom the absorbed dose to the kidneys reached 23 Gy showed a significantly longer survival (median OS 60 months) than those in whom it did not (median OS 19 months, *p* < 0.0006; Fig. 4). The majority of patients in whom the dose to the kidneys did not reach 23 Gy had progression or bone marrow suppression. Still, the comparison implies that a cut-off defined by four cycles of 7.4 GBq will exclude patients who might benefit from further therapy. This view is supported by the finding that differences in survival between the patient groups could still be observed when patients who stopped treatment because of tumour progression or deterioration for other reasons were excluded. In the remaining patients therapy was stopped due to kidney dosimetry, slow bone marrow recovery without other complications, decrease in tumour burden and the patient’s wish to stop after four cycles (Table 3).

Although more than half of our patients had bone metastases, the morphological response rate was similar or superior to that in other studies (Table 7) and the subacute haematotoxicity was not higher in this group (Table 3). Unlike in other studies, the diagnosis of bone and bone marrow metastases was based on posttreatment scans during therapy, which has to be regarded as more sensitive than somatostatin receptor scintigraphy, and this may be one possible explanation for this high figure. Although in none of our patients was an absorbed dose of 2 Gy to the bone marrow reached, 22% had to stop therapy due to bone marrow-related events.

In agreement with others [22, 30, 40], we found a considerable interindividual variation in calculated bone marrow doses, and similar median values in patients with and without therapy-limiting bone marrow toxicity (Table 3). Although a correlation between absorbed dose and bone marrow toxicity has previously been shown by applying a dosimetry method based on planar imaging supported with SPECT/CT [42], our finding emphasizes that bone marrow dosimetry at the current state of our knowledge cannot be used to predict bone marrow toxicity in the individual patient. In line with the view of these authors, both their method and the method used in the current study will underestimate bone marrow doses in patients with bone marrow metastases. Also, the interindividual variation mentioned above will contribute to uncertainty in predicting toxicity.

The incidence of bone marrow malignancies in four patients (2%) is in line with reports from other centres [43]. Of previously documented risk factors, all had bone metastases and one had extensive liver metastases. Three patients (75%) had received the alkylating agent temozolomide, and one had also received streptozotocin. In this series, temozolomide is over-represented among patients with bone marrow malignancies despite the fact that streptozotocin (in combination with 5-fluorouracil) was the predominant alkylator used in 23.9% of all patients compared with 15.3% who had received temozolomide. This finding is an isolated observation and must be interpreted with caution. Still, as a comparatively new agent in the therapeutic arsenal of NETs, this toxicity should be observed more closely. As discussed by Bodei et al. [43], bone marrow malignancy is a fairly infrequent finding in larger PRRT series, even though one recent study showed a much higher incidence of bone marrow malignancies in a small group of heavily pretreated patients (20%, 4 of 20 patients) [44]. The present study, in line with several others, showed favourable tumour responses and survival data that compare well with alternative treatments. Especially in rectal NETs and bronchopulmonary carcinoids [23], the outcome is most encouraging.

In our opinion, this study provides important findings in favour of an individualized, dose-driven PRRT protocol. A prospective randomized study comparing the treatment protocol involving four cycles of 7.4 GBq of ^177^Lu-DOTA-octreotate with a protocol based on kidney dose might answer the question as to whether the observed superior outcome in patients in whom the absorbed dose to the kidneys reached 23 Gy was a result of optimization of the tumour dose or depended on individual patient and tumour characteristics.

### Conclusion

Dosimetry-based planning of therapy with ^177^Lu-DOTA-octreotate is feasible. In the majority of patients (61.5%) the intended absorbed dose of 23 Gy to the kidneys was reached after three to nine cycles of 7.4 GBq ^177^Lu-DOTA-octreotate. In no patient was a cumulative dose of 2 Gy to the bone marrow reached. Bone marrow toxicity was in line with previous reports even though the majority of patients had bone marrow metastases and/or advanced disease. No major nephrotoxicity entirely related to the treatment was found, but 4% of patients developed grade 2 toxicity at the time of progression, and one patient (0.5%), later diagnosed with arteriosclerotic nephrosclerosis, developed grade 4 toxicity without the dose limit of 23 Gy to the kidneys having been reached. Patients in whom this absorbed dose was reached, had significantly longer PFS and OS, and significantly more patients obtained CR/PR as well as biochemical responses. Patients with CR/PR survived longer than those with SD.
